# Spatial and temporal relationships between human and canine visceral leishmaniases in Belo Horizonte, Minas Gerais, 2006–2013

**DOI:** 10.1186/s13071-018-2877-6

**Published:** 2018-06-28

**Authors:** Fábio Raphael Pascoti Bruhn, Maria Helena Franco Morais, Denis Lucio Cardoso, Nádia Campos Pereira Bruhn, Fernando Ferreira, Christiane Maria Barcellos Magalhães da Rocha

**Affiliations:** 1 Federal University of Pelotas (UFPel), Department of Preventive Veterinary, Zoonosis Control Center of UFPel, Capão do Leão, Rio Grande do Sul Brazil; 2 Municipal Department of Health of Belo Horizonte, Belo Horizonte, Minas Gerais Brazil; 3 Department of Preventive Veterinary, Federal University of Lavras (UFLA), Belo Horizonte, Minas Gerais Brazil; 4 Center of Mercosul Integration, Federal University of Pelotas (UFPel), Pelotas, Rio Grande do Sul Brazil; 5 Department of Preventive Veterinary Medicine and Animal Health, School of Veterinary Medicine, University of São Paulo, São Paulo, Brazil

**Keywords:** *Leishmania infantum*, Public health, Spatial analysis, Time series, Epidemiology

## Abstract

**Background:**

Visceral leishmaniasis is a serious public health problem in Brazil, and control of this disease constitutes a major challenge. The purpose of this study was to assess the existing spatial and temporal relationships between cases of canine visceral leishmaniasis (CanL) and human visceral leishmaniasis (HVL) recorded in Belo Horizonte, State of Minas Gerais, from 2006 to 2013.

**Methods:**

Data provided by the Belo Horizonte Health Services regarding the disease control routine were analyzed in order to perform a retrospective observational and ecological study. Information regarding the incidence rate of HVL and canine seroprevalence was examined in relation to control actions performed atthe 148 coverage areas of healthcare centers for the period between 2006 and 2013. A time series analysis was performed using the Gretl 1.9.12 software followed by the assessment of the existing increasing or declining trend and seasonality in the occurrence of CanL and HVL. Autoregressive integrated moving average (ARIMA) models were adjusted, intervention analysis was performed, vector autoregressive models were developed, and Granger causality was used for testing temporal relationships between variables. The hot spot analysis tool was used for cluster identification through Getis-OrdGi statistics. The ArcGis for desktop 10.2.1 software was used for spatial analysis.

**Results:**

We identified 866 HVL cases in Belo Horizonte between 2006 and 2013. The mean proportion of canine seroprevalence (PCP) was 7.31% and the mean proportion of monitored hosts (PMH) was 6.73%.HVL and PCP showed a decreasing trend, while PMH increased over time (*P*<0.05). Vector Autoregressive (VAR) and Granger analysis showed a temporal relation between CanL and HVL cases. Maps illustrating the spatial distribution of cases and obituaries of HVL and CanL cases also showed an apparent association between the occurrence of leishmaniasis in humans, and data about canine cases recorded in the previous years.

**Conclusions:**

Cases of HVL were preceded by PMH and PCP cases. Similar results were observed for intraspecific cases (i.e. between PCP and other canine cases and between HVL and other HVL cases), which indicated the existence of favorable environmental conditions for the transmission and spread of *L. infantum* in Belo Horizonte.

## Background

Visceral leishmaniasis (VL) is a major neglected zoonotic disease, an infectious chronic and systemic disease, which may reach 90% mortality if not treated [1]. Despite high mortality, the majority of infections caused by *Leishmania infantum* in humans are asymptomatic. However, people living in conditions of social vulnerability are more susceptible to the development of clinical signs [2–6], which may account for the fact that although leishmaniasis has been identified in 98 countries, most cases of human visceral leishmaniasis (HVL) are found in Brazil, Bangladesh, Ethiopia, India, Sudan and South Sudan [2].

VL is a serious public-health problem in Brazil [7]. A total of 74,980 new cases were identified between 1990 and 2013, with a mean annual incidence of 3124 cases, corresponding to a mean incidence rate of 1.8 patients per 100,000 inhabitants [8].

One cause of the increase in HVL morbidity is the difficulty of Municipal Health Services to execute the resolutions of the Surveillance Program and Visceral Leishmaniasis Control (SP-VLC). Thus, considering the impact of leishmaniasis in the country, development of novel ways to control VL, and assessment and improvement of current approaches is required. However, this improvement depends on the epidemiological characteristics of the disease such as spatial and temporal distribution, and association between variables. These parameters are important because they help to understand the health-disease process and the implementation of educational, prophylactic, and targeted control measures, as well as the implementation of monitoring and surveillance practices.

HVL in the municipality of Belo Horizonte has been observed since 1994. Since then, healthcare services have been structured in a pioneering way in order to record data regarding disease control measures. Priority areas regarding disease occurrence were identified based on measures developed to improve the knowledge about the importance of spatial distribution, in order to optimize human and financial resources, and to reduce the negative impact of VL.

The recommendation of euthanasia for seropositive dogs is one control measure of VL adopted in Belo Horizonte. Although this measure alone may not be sufficient [9, 10], euthanasia is still considered in the Brazilian legislation as a method of disease control in the country.

Considering the importance of promoting VL control in endemic areas in Brazil and the challenges of euthanasia of canine reservoirs, we aimed to assess the existing spatial and temporal relationships between cases of canine visceral leishmaniasis (CanL) and HVL recorded in different administrative regions in the municipality of Belo Horizonte, State of Minas Gerais, from 2006 to 2013. Here, we hypothesized that HVL occurrence in an endemic area that promotes the euthanasia of dogs as a control measure may change over the years and over space. In addition, we examine whether the presence and maintenance of canine reservoirs in the urban environment may precede human cases in time and space in a VL endemic area such Belo Horizonte.

## Methods

Data regarding VL control in Belo Horizonte, State of Minas Gerais, were analyzed in order to perform a retrospective observational and ecological study. Information referring to the incidence rate of HVL and obituaries was assessed. In addition, the seroprevalence of CanL and the proportion of monitored hosts between 2006 and 2013 were investigated.

### Site characterization

Belo Horizonte is the sixth largest city in Brazil and the capital of the State of Minas Gerais. It consists of a population of 2,479,165 inhabitants distributed in an area of 331.4 km^2^, with a population density of 7167 inhabitants/km^2^ estimated in 2013. The majority of the population (91%) in the city is educated, the gross national product per capita (according to 2011 statistical data) was approximately US$ 7136.29 (R$23,053.07), and the basic sanitation coverage is about 96.1% [11].

The city is located at the following geographical coordinates: 19°49'01"S, 43°57'21"W and 852 m elevation. It is characterized by a dry winter and rainy summer, with 21°C annual mean temperature, 65% relative humidity and 1500 mm average precipitation.

**Fig. 1:**
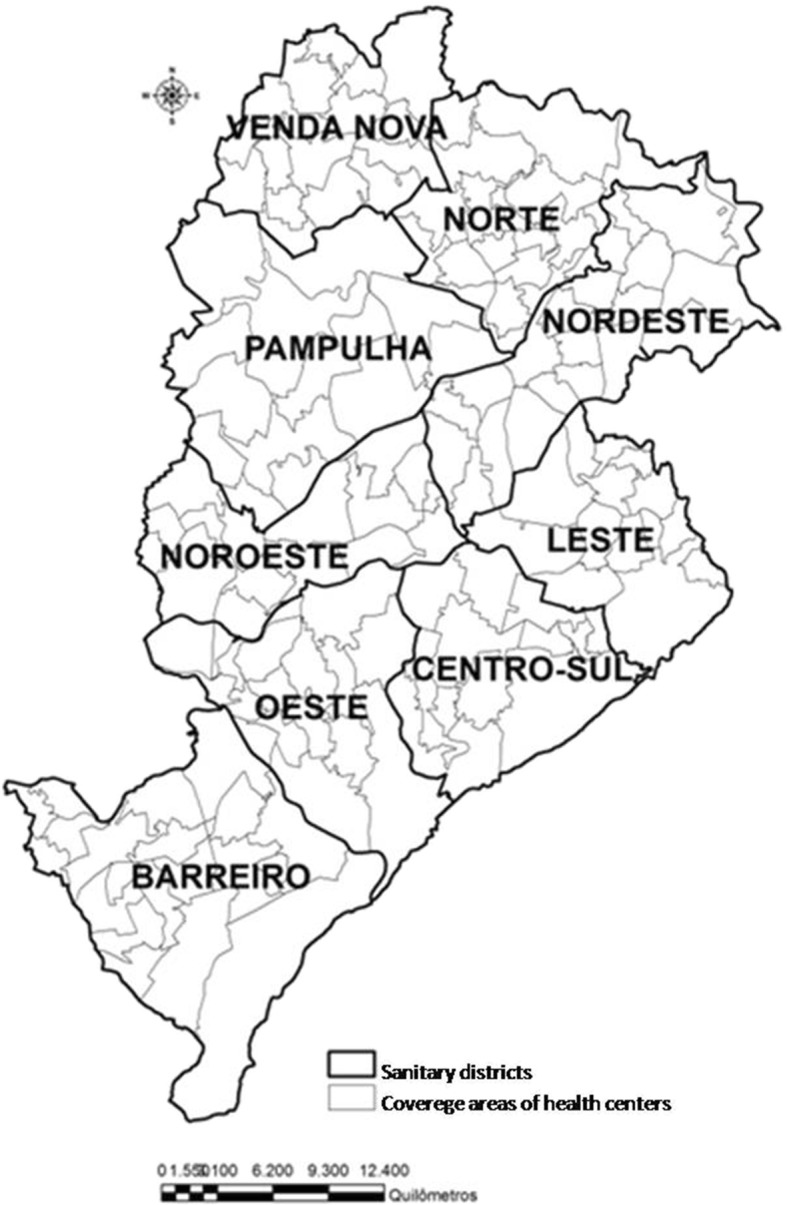
Coverage area of nine Sanitary Districts of Belo Horizonte, Minas Gerais, Brazil

The Belo Horizonte health service is organized into nine Sanitary Districts (SD) categorized based on demographic statistics, habitation, education and health. These SDs comprise the health administrative regions of Barreiro, South-Central, East, Northeast, Northwest, North, West, Pampulha and Venda Nova, and are divided into 148 coverage areas of Healthcare Centers (Fig. 1). This administrative division was based on the following criteria: sets of census sectors, existence of geographical barriers and aggregation, transportation, and homogeneity of characteristics. In addition, 328 healthcare facilities exist in these areas, which belong to the Brazilian Unified Health System (SUS) and care for an average population of approximately 15,331 inhabitants (ranging from 2197 to 45,171, depending on the area) [12].

### Data collection

Data regarding CanL and HVL cases, as well as deaths caused by HVL, were georeferenced and clustered in different coverage areas of healthcare facilities for data analysis. Data regarding VL control actions, provided by the Management of Epidemiology and Information (GEEPI) and Zoonosis Control Management (GECOZ) of the Municipal Health Department (MHD) of Belo Horizonte, were also analyzed.

Data used in this study referred to canine seroreactivity, and were linked to actions against VL between 2006 and 2013. The following variables were found in the Information System for Zoonoses Control (SCZOO): (i) identification (address and name of the owner) of dogs examined through census inquiries; (ii) laboratory test results of these dogs; (iii) spatial distribution of the examined dogs; and (iv) history of CanL seropositivity. The Information System of the MHD of Belo Horizonte was used for georeferencing data regarding dogs’ residence, provided by the Data Processing Company of the Municipality of Belo Horizonte (PRODABEL).

To appreciate canine seroprevalence and perform reservoir control, areas with HVL and prevalence of canine seroreactivity above 2% should be subject to annual canine serological census for at least three consecutive years [8]. Thus, in Belo Horizonte, the average proportion of the areas with census canine surveys between 2006 and 2013 was 98.6% and the average proportion of the canine population that was investigated through serological diagnostic methods in the survey was 48.7% [13]. These results indicate high representation of the canine surveys among areas with transmission of *L. infantum* and in the canine resident population in the municipality. The collection and processing of data regarding laboratory tests were performed as outlined in the protocol of the Ministry of Health between 2006 and 2013 [enzyme-linked immunosorbent assay (ELISA) techniques for screening and indirect immunofluorescence test (IIFT) for data confirmation]. Tests were performed in the Laboratory for Zoonoses Control of the MHD of Belo Horizonte.

### Processing and data analysis

Data were analyzed using temporal and spatial statistical methods aiming to describe the relationship between cases of CanL and HVL recorded in Belo Horizonte, State of Minas Gerais, from 2006 to 2013.

Analyses of variables related to human and animal morbidity due to infection were performed. In addition, the relationship between temporal series under study was assessed using temporal and spatial statistical methodology. The effect of canine reservoir hosts on HVL was interrogated by developing indicators related to the disease morbidity on dogs:

(i) Proportion of canine seroprevalence (PCP):

$$ \mathrm{PCP}=\frac{\mathrm{Seropositive}\ \mathrm{dogs}\ \mathrm{by}\ \mathrm{the}\ \mathrm{ELISA}\ \mathrm{and}\ \mathrm{IIFT}}{\mathrm{Total}\ \mathrm{assessed}\ \mathrm{cases}} $$

(ii) Proportion of monitoring hosts (PMH)

$$ \mathrm{PMH}=\frac{\mathrm{Seropositive}\ \mathrm{dogs}\ \mathrm{by}\kern0.1em \mathrm{the}\ \mathrm{ELISA}\ \mathrm{test}\ \mathrm{and}\ \mathrm{negativeor}\ \mathrm{indeterminate}\ \mathrm{result}\ \mathrm{by}\ \mathrm{the}\ \mathrm{IIFT}}{\mathrm{Total}\ \mathrm{assessed}\ \mathrm{cases}} $$

These indicators were constructed due to their use in the VL control practice in Belo Horizonte, in relation to dogs’ euthanasia. PCP represents the proportion of dogs tested positive in both ELISA and IIFT diagnostic tests, therefore those animals were recommended for euthanasia, in compliance with the Brazilian legislation. PMH represents those animals that were seropositive according to ELISA but were negative according to IIFT, therefore they were not recommended for euthanasia and thus remained in the environment. These animals are monitored annually by the MHD.

### Time series

The incidence of HLV, PCP and PMH estimated for the period between 2006 and 2013 was analyzed. Time series were analyzed using the Gretl 1.9.12 statistical software (GNU Regression, Econometric and Time-series Library) to determine temporal associations and/or seasonality (occurrence relative to seasons), as well as for autoregressive integrated moving average (ARIMA) modeling (ARIMA is a type of temporal series modeling, which in this study was construed as a means to apply other types of tests, such as intervention and vector autoregressive models). In addition, intervention analysis (evaluation of the presence of statistically significant peaks in the occurrence of the series at any month over the years) was performed for a better exploratory characterization of time series components. Vector autoregressive (VAR) models and Granger causality tests were performed to test the dynamic relationships between variables under study over time.

Thus, the existence of the assumptions for the application of the tests mentioned above was evaluated, specifically the stationarity (required for ARIMA modeling, Granger and VAR causality tests) and the presence of two co-integration vectors (required for VAR modeling). It is important to highlight that the stationarity concept is an important assumption considered in the time series modeling process, i.e. the series should develop over time, around an average and constant variance, reflecting a form of a stable equilibrium.

In this study, the assessment of the time series stationarity was performed using the Augmented Dickey-Fuller (ADF) testing; data visualization on correlogram; assessment of the suitability of the model proposed using Akaike information criterion (AIC), Bayesian information criterion (BIC) or Schwarz criterion; and Box and Pierce test (Q < *χ*^2^). The assessment was also performed through the residual visualization on correlogram [14].

We assumed that variables included in the model are stationary, especially for VAR analysis and Granger causality testing, which assessed the relationship between the proportion of canine seroprevalence or the proportion of monitored hosts and HVL incidence. Using the ADF test, we identified that the series of HVL, PCP and PMH are non-stationary, which became stationary after adding the first difference to the original series. This was done because the regression of a non-stationary time series may result in a spurious regression in comparison with another non-stationary time series [15].

In the VAR and Granger modeling process, the number of lags on the model was estimated using the lags selection method of vector autoregressive models before Johansen cointegration tests. This estimate was acquired by the number of months during which the previous independent variable, which would have an effect on dependent variables, occurred. The selection of lags was performed based on AIC, BIC or Schwarz criterion, and Hannan-Quinn criterion (HQC). In this study, lags containing two periods were selected based on results found for these criteria, while the presence of two cointegration vectors for each pair of variables under study were shown using Johansen cointegration tests. Thus, the null hypothesis regarding the existence of two cointegration vectors was rejected, suggesting the existence of two cointegrating relationships for the proposed model. This result suggests the suitability of the VAR model, once the number of cointegration vectors was equal to the number of variables under study [15].

### Spatial analysis

The ground system of geographical coordinates was used for spatial analysis, according to ordered pairs of coordinates (x, y) for examined dogs, cases of HVL and deaths by HVL.

Thematic maps referring to the distribution of cases of CanL and HVL in the coverage areas of healthcare facilities of Belo Horizonte were developed for exploratory spatial data analysis. We used shapefiles provided by the MHD of Belo Horizonte, the spatial data storage format, which consists of the position, shape and attributes of geographical features. Maps were annually designed based on the overlapping of HVL cases with CanL cases counted in each coverage area. Classes referring to the occurrence of CanL cases were resolved according to the distribution of CanL cases and monitored hosts over the years.

The hot spot analysis tool was used for the identification of spatial clustering *via* Getis-OrdGi statistics, which identifies spatial clusters of high values (hot spots) and spatial clusters of low values (cold spots) [16].

According to Getis & Ord [17], the hot spot analysis calculates the concentration or the lack of concentration of the sum of the values associated with variables in a dataset. Assuming a normal distribution pattern, when the probability associated with a *z*-score is positively or negatively greater than a specific significance level (*P*-value), it obtains a positive or negative spatial association. Thus, for positive *z*-scores, the intensity of spatial clustering of high values (hot spots) increases when the *z*-score is higher, and for negative *z*-scores, the intensity of spatial clustering of low values (cold spots) increases when the *z*-score is lower.

## Results

The epidemiological indicators of HVL and CanL in Belo Horizonte are presented in Table 1. We identified 866 HVL cases in Belo Horizonte between 2006 and 2013. Based on the evaluation of the distribution of HVL cases among health districts, we verified that the highest HVL incidence was observed in the Northeast, Venda Nova, Northwest and North districts. In addition, HVL incidence appeared to decline over the years. Similarly, PCP showed higher values in the Northeast and Venda Nova districts, in addition to an apparent decline trend over the years, from 11.91 ± 3.93% in 2006 to 3.54 ± 0.40% in 2013. Time series modeling confirmed that the decrease in HVL incidence and PCP were statistically significant (*P* < 0.05). In contrast PMH increased over time (*P* < 0.05). Indicators of morbidity for CanL and HVL showed a trend over time (*P* < 0.05); however, no seasonality was observed (*P* > 0.05).

**Table 1:** HVL, PCP (%) and PMH (%) in Belo Horizonte, Minas Gerais, Brazil, 2006–2013 broken down by year and sanitary district

Variable		HVL	CanL	
		Cases	PMH	PCP
Sanitary district	Barreiro	77	8.3	6.8
	Centro-sul	36	5.2	3.4
	Leste	94	5.4	6.0
	Nordeste	156	7.3	7.5
	Noroeste	148	5.5	6.6
	Norte	96	6.2	6.7
	Oeste	74	5.9	5.8
	Pampulha	45	6.9	6.8
	Venda nova	133	6.2	7.9
	Total^a^	866	6.3	6.4
Year^b^	2006	128	11.9 ± 3.9	4.8 ± 1.4
	2007	111	10.4 ± 3.6	3.7 ± 2.4
	2008	160	7.5 ± 1.9	6.0 ± 4.9
	2009	141	7.7 ± 3.8	12.1 ± 2.7
	2010	131	7.9 ± 1.3	9.3 ± 2.7
	2011	94	6.5 ± 3.6	6.9 ± 2.7
	2012	59	3.1 ± 0.7	4.1 ± 1.0
	2013	42	3.5 ± 0.4	6.9 ± 3.7
	Total	866	7.31	6.73

*Abbreviations*: *HVL* human visceral leishmaniasis, *CanL* canine leishmaniasis, *PCP* proportion of canine prevalence = positive dogs in ELISA and RIFI/ dogs evaluated, *PMH* proportion of monitoring hosts (dogs not euthanized by the health service) = positive dogs only in the ELISA/ dogs evaluated

^a^In seven cases presented in the database, the sanitary district of origin was ignored

^b^Until July2013; PMH and PCP: mean ± standard deviation

Table 2 shows the results obtained after ARIMA modeling and intervention analyses in the HVL, PCP and PMH indicators. Intervention analysis in human cases, based on the adjustment of the ARIMA model, showed statistically significant peaks for HVL cases in January 2008, with lags consisting of 3 and 4 periods (*P* < 0.01). This result suggested an intervention in the series once, after this period the pattern of the series had changed, showing a decreasing trend in the following months.PCP showed a statistically significant peak that occurred in January 2011, with lags consisting of 1 and 2 periods (*P* < 0.01), and suggested a possible intervention in the series once, after this period the pattern of the series had changed, showing a decreasing trend.

**Table 2:** Time series and intervention models for HVL, PCP (%) and PMH (%) in Belo Horizonte, Minas Gerais, Brazil, 2006–2013

Indicator	Variable	Coefficient	SE	*Z*-value	*P*-value
HVL^a^	Theta_1	-1.03	0.05	-19.53	< 0.0001
	Theta_5	-0.37	0.11	-3.33	< 0.0001
	Theta_6	0.55	0.09	5.67	< 0.0001
	Dummy_jan_2008_3	0.28	0.09	3.09	< 0.0001
	Dummy_jan_2008_4	-0.30	0.00	-3.36	< 0.0001
PCP^b^	Phi_1	0.62	0.08	7.20	< 0.0001
	Theta_1	-1.00	0.06	-14.94	< 0.0001
	Dummy_jan_2011	0.68	0.24	2.82	< 0.0001
	Dummy_jan_2011_1	-1.83	0.43	-4.19	< 0.0001
	Dummy_jan_2011_2	1.13	0.25	4.49	< 0.0001
PMH^c^	Phi_1	0.99	0.07	12.61	< 0.0001
	Phi_3	-0.18	0.08	-2.25	0.02
	Theta_1	-1.49	0.03	-39.96	< 0.0001
	Theta_3	0.49	0.03	13.19	< 0.0001
	Dummy_apr_2008	0.76	0.06	11.42	< 0.0001
	Dummy_aug_2009	-6.87	1.36	-5.04	< 0.0001
	Dummy_aug_2009_1	5.97	1.31	4.53	< 0.0001

*Abbreviations*: *HVL* human visceral leishmaniasis, *PCP* proportion of canine prevalence = positive dogs in ELISA and RIFI/ dogs evaluated, *PMH* proportion of monitoring hosts (dogs not euthanized by the health service) = positive dogs only in the ELISA/ dogs evaluated; *SE* standard error

^a^HVL: Autoregressive integrated moving average model (ARIMA) (0, 1, 6) adjusted; Akaike information criterion: 103.40, Schwarz criterion: 118.19, and Hannan-Quinn criterion: 109.35

^b^PCP: ARIMA (1, 1, 1) adjusted; Akaike information criterion: 25.99, Schwarz criterion: 40.93, and Hannan-Quinn criterion: 32.01

^c^PMH:ARIMA (3, 1, 3) adjusted; Akaike information criterion: 425.40, Schwarz criterion: 445.40, and Hannan-Quinn criterion: 433.46

The analysis of the intervention in the time series of PMH (Table 2) showed a statistically significant (*P* < 0.01) decrease that occurred in April 2008, followed by an expressive increase, and then a peak occurred in August 2009 (*P* < 0.01). This peak suggested a possible intervention in the series once, after this period the pattern of the series had changed, showing a decreasing trend.

### PCP and HVL incidence

VAR analysis was performed to assess the temporal relationship between PCP and HVL (Table 3). This model indicated that cases of HVL are preceded by cases of PCP that had occurred approximately two months before the records of HVL (*P* = 0.03). In addition, an intraspecies relationship was also found, since the occurrence of CanL cases also influences the occurrence of new CanL cases in the city.

**Table 3:** Dynamic relationship (VAR) between PCP (%)for CanL and incidence of HVL, Belo Horizonte, Minas Gerais, Brazil, 2006–2013

Independent variables	Dependent variables			
	HVL		PCP	
	Coefficient	*P*-value	Coefficient	*P*-value
HVL_1	-0.67	< 0.0001	-0.09	0.24
HVL_2	-0.35	< 0.0001	-0.03	0.68
PCP_1	-0.07	0.58	-0.31	< 0.0001
PCP_2	0.27	0.03	-0.18	0.09
*R* ^2^	0.39		0.11	
Adjusted *R*^2^	0.37		0.08	
Durbin-Watson	2.08		1.99	

*Abbreviations*: *VAR* vector autoregressive model, *HVL_1* human cases recorded a month later, *HVL_2* human cases recorded two months later, *PCP_1* proportion of canine seroprevalence recorded a month later, *PCP_2* proportion of canine seroprevalence recorded two months later, *HVL* human visceral leishmaniasis, *CanL* canine leishmaniasis; *PCP* proportion of canine prevalence = positive dogs in ELISA and RIFI/ dogs evaluated

Regarding the Granger causality testing, our results indicate that cases of HVL are preceded by cases of PCP (*P* = 0.05). Similar results were observed between PCP and other canine cases (*P* = 0.01). However, cases of PCP are not preceded by HVL cases in Belo Horizonte (*P* = 0.49).

### PMH and HVL incidence

Table 4 shows the results of the analysis of the temporal relationship between PMH and HVL regarding the VAR model. This model indicated that cases of HVL are preceded by cases of PMH that occurred approximately two months before the records of HVL (*P* = 0.04).

**Table 4:** Dynamic relationship (VAR) between PMH (%) for CanL and incidence of HVL, Belo Horizonte, Minas Gerais, Brazil, 2006–2013

Independent variables	Dependent variables			
	HVL		PMH	
	Coefficient	*P*-value	Coefficient	*P*-value
HVL_1	-0.67	< 0.0001	0.07	0.35
HVL_2	-0.30	< 0.0001	0.12	0.11
PMH_1	-0.14	0.31	-0.24	0.02
PMH_2	0.30	0.04	-0.19	0.08
*R* ^2^	0.40	0.13		
Adjusted *R*^2^	0.38	0.09		
Durbin-Watson	2.10	2.04		

*Abbreviations*: *VAR* vector autoregressive model, *HVL_1* human cases recorded a month later, *HVL_2* human cases recorded two months later, *PMH _1* proportion of monitoring hosts recorded a month later, *PMH _2* proportion of canine monitoring hosts recorded two months later, *HVL* human visceral leishmaniasis, *CanL* canine leishmaniasis, *PMH* proportion of monitoring hosts (dogs not euthanized by the health service) = positive dogs only in the ELISA/ dogs evaluated

The Granger causality testing indicate that cases of HVL are preceded by cases of PMH (*P* = 0.03). However, according to the model, cases of PCP are not preceded by HVL cases in Belo Horizonte (*P* = 0.27).

### Spatial analysis

**Fig. 2:**
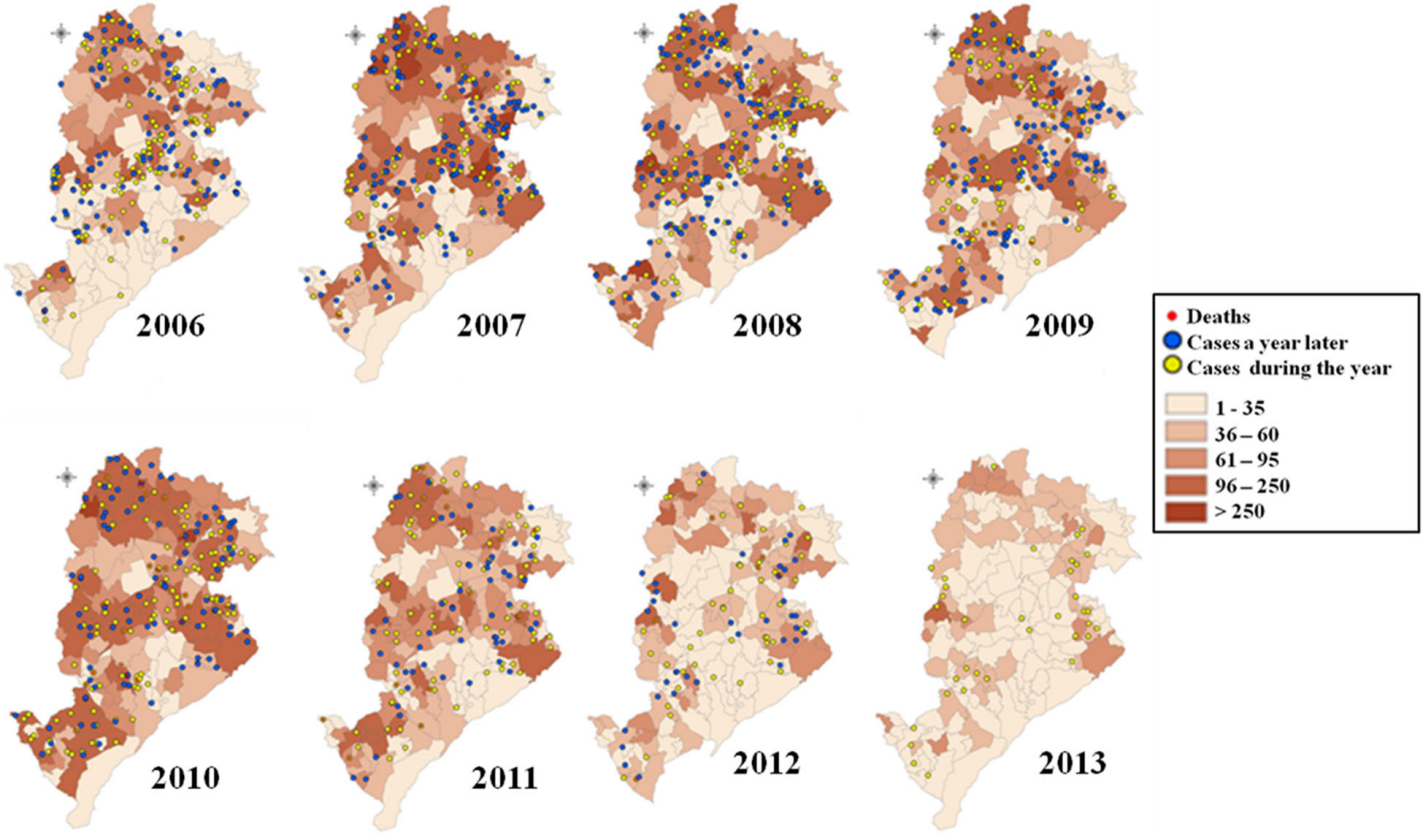
Distribution of canine leishmaniasis (CanL) and human visceral leishmaniasis (HVL) cases and deaths in Belo Horizonte, Minas Gerais, Brazil, 2006–2013. Cases of canine seropositivity according to the ELISA test and IIFT

**Fig. 3:**
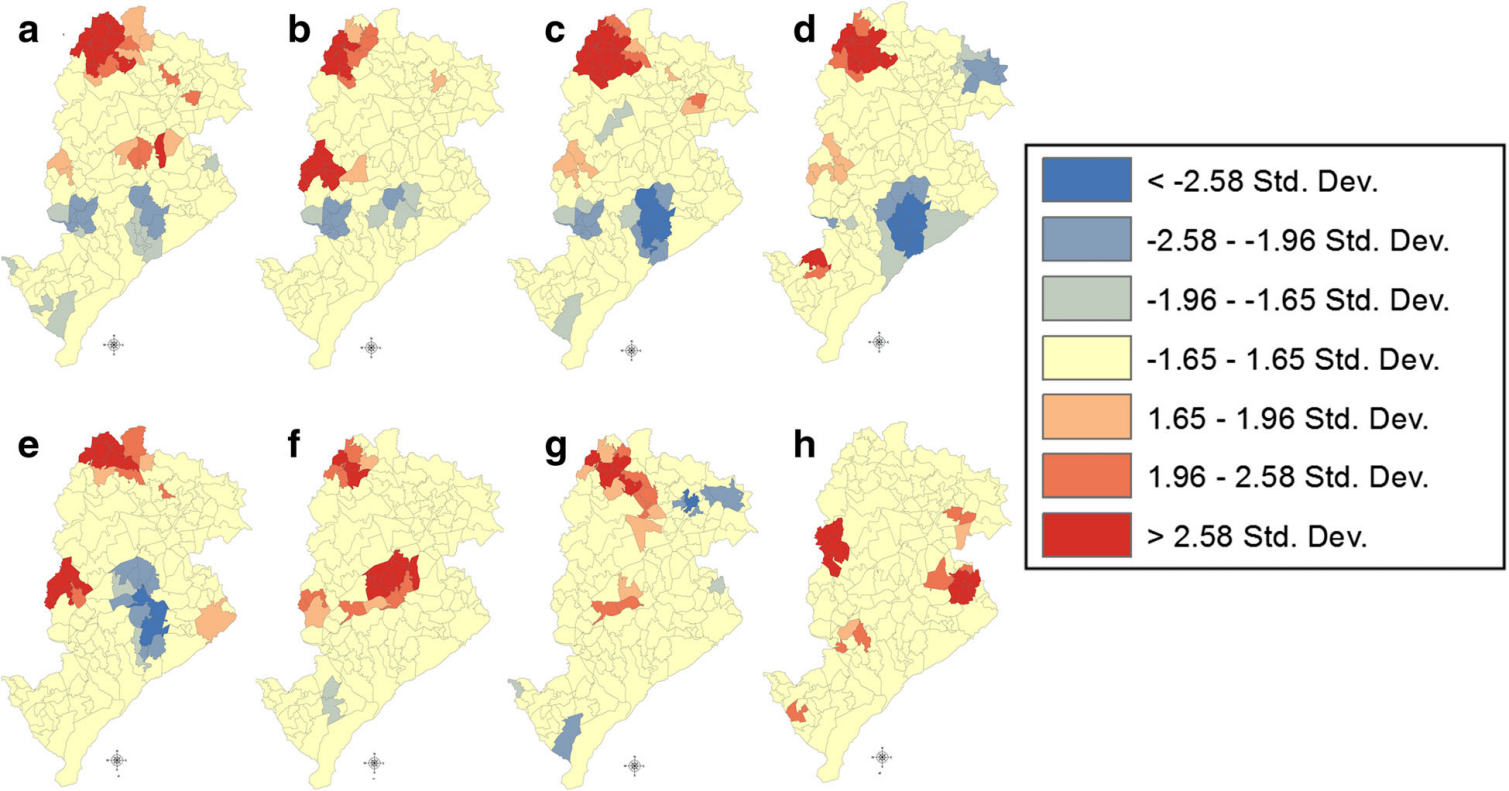
Areas containing clusters (hot spot analysis) of canine leishmaniasis (CanL) and human visceral leishmaniasis (HVL), Belo Horizonte, Minas Gerais, Brazil, 2006 to 2013.Hot spot analysis was performed with the ArcGis 10.2.1 software. Red areas indicate clusters of high values (hot spots); blue areas indicate clusters of low values (cold spots). CanL cases according to the ELISA test and IIFT. **a** CanL cases recorded in 2007. **b** CanL cases recorded in 2008. **c** CanL cases recorded in 2010. **d** CanL cases recorded in 2011. **e** CanL cases recorded in 2013. **f** HVL cases recorded in 2006. **g** HVL cases recorded in 2009. **h** HVL cases recorded in 2013

Figure 2 illustrates the thematic maps used for the exploratory spatial data analysis. This analysis allowed for the identification of spatial patterns, which were statistically confirmed through hot spot analysis (Fig. 3). This analysis revealed the spatial cluster maps for HVL and CanL cases of the regions under study, during the years that showed a statistically significant clustering (*P* < 0.05).

Maps illustrating the spatial distribution of cases and obituaries of HVL and CanL (Fig. 2) described an apparent association between the occurrence of HVL and CanL recorded in previous years. This pattern is observed on both spatial distribution maps (Fig. 2) and spatial cluster maps (Fig. 3). Figure 2 also illustrates that HVL and CanL cases are mainly concentrated along the coverage areas of SDs of Venda Nova, North, East, Northeast and Northwest, forming a band or area of high incidence of HVL and CanL cases during the study period.

There is a change in respect to the spatial distribution of HVL cases in relation to CanL cases, especially in 2009. HVL cases appear to be moving away from areas containing greater spatial concentration of CanL towards areas containing an expressive increase of incidence of PMH cases, mainly from 2008 (Fig. 2).

## Discussion

### Time series

Indicators related to the disease morbidity and quality of health services in regions under study showed a trend over time; however, they showed no seasonal variation (*P* < 0.05). Proportions of HVL cases and CanL seroprevalence showed a decreasing trend, while PMH increased over time. This pattern may represent a high epidemiological risk, because PMH of *L. infantum*, which are not euthanized, may be important sources of infection for both vectors and humans. The relation between PMH and HVL was confirmed in our study by both temporal and special analyses.

The seroprevalence of CanL is high worldwide. Mean CanL seroprevalence in our study was 6.73%, which was below the one estimated by Khanmohammadi et al. [18] in Iran (8.5%). Dujardin et al. [19] reported that CanL seroprevalence in Europe, among endemic countries, was close to 25%. A similar situation has already been discussed by Franco et al. [20], who discovered high variability (< 5% to > 30%) in the distribution of CanL in European countries between 1971 and 2006. These authors speculated that variations in the occurrence of visceral leishmaniasis depended essentially climate, ecological and socioeconomic characteristics that favor the transmission of diseases more than the simple presence of etiologic agents or seropositive dogs in the environment.

A study performed by Brito et al. [1] in Jaciara, State of Mato Grosso, from 2003 to 2012, showed that individuals were most commonly affected during the May-July period, which follows a period of greater density of vectors in the environment, suggesting high seasonal variation. Mestre &Fontes [21] also performed a study in the same Brazilian State and found that the disease transmission did not show a seasonal pattern due to the variation of the length of the incubation period and the variation in the time spent post-infection to seek for medical treatment.

According to Colla-Jacques et al. [22], in Brazil, rainfall and temperature are the two main factors that determine soil moisture, which influences the quality of organic matter used by *L. longipalpis* to deposit eggs. Seasonality is a spatial distribution characteristic of *L. longipalpis* and consequently of HVL and CanL cases [22, 23]. Thus, knowing the periods of greater occurrence of vectors is critical for the implementation of control measures. However, the lack of well-defined seasons in the Southeast region of Brazil, for example in Belo Horizonte, leads to difficulties in establishing seasons of vector abundance, mainly in studies that performed data collection for short periods of time. In Tunisia, a temperate region, Benabid et al. [23] established that the period of greatest occurrence of vectors occurred between April and November, therefore parasite transmission is more likely during this period.

Our intervention analysis identified peaks that probably reflect interventions, which could be partially explained by changes made to the visceral leishmaniasis control program in Belo Horizonte over the years. Some of these changes were described by Morais et al. [13], who assessed the effectiveness of control measures applied in the municipality of Belo Horizonte, between 2006 and 2011. In our study, a peak was found regarding the incidence of HVL in January 2008, followed by a decreasing trend. This situation may be related to the improvement of the annual operating capacity of vectors control, which varied from 170,000 in 2007 to 189,000 in 2008. In addition, the number of seropositive dogs recorded in census inquiries varied from 3110 in 2006 to 8296 in 2007 [13].

Other peaks were found in relation to PCP and PMH. A PCP peak was found in January 2011, followed by a decreasing trend in the next months. This epidemiological change may be explained by changes made to the structure of the visceral leishmaniasis control program in Belo Horizonte between 2009 and 2010, with effect on the effectiveness of visceral leishmaniasis control measures that were adopted in the municipality. Thus, these changes were evaluated by Morais et al. [13] through the following indicators: productivity of blood sample collection teams in dogs (65.7–84.3%), use of available tests (71.4–91.7%), coverage of areas with canine census inquiries (94.6–119.5%) and coverage of canine population with census inquiries (41.5–60.5%). Morais et al. [13] found that the productivity of blood sample collection teams increased from 2009 to 2010, especially in relation to the control actions applied to reservoirs hosts, by improving the use of existing resources, geographical expansion of actions and, consequently, better suitability for the guidance of the SP-VLC.

A significant decrease in PMH (*P* < 0.05) was recorded in April 2008, whereas an increase, marked by a peak, was recorded in August 2009 (*P* < 0.05), followed by a decreasing trend. Morais et al. [13] speculated that the variations recorded in relation to HVL prevalence may be partially explained based on the following: (i) expansion of the areas under census inquiries, which followed the direction of the infection spread in the municipality; (ii) adoption of susceptible and/or infected dogs by the local residents, in order to replace dogs taken by animal health control agents; and (iii) variation on the serological test sensitivity. It is important to clarify that these variations occurred between 2009 and 2010 due to the lack of reproducibility of the IIFT test in the daily routine, and of appropriate quality controls in the Laboratory for Zoonoses Control of the Municipal Health Department of Belo Horizonte. Considering the limitations of the current diagnosis protocol, the Ministry of Health provided a new protocol for use since 2011. However, the implementation of the protocol in the municipality of Belo Horizonte started in 2013 [13].

VAR analysis and Granger causality testing described the temporal relationship between PCP/PMH and HVL. These tests showed a significant relationship between CanL with HVL cases (*P* < 0.05). As presented above, PCP represents the proportion of dogs tested positive in the multiple series test used for CanL diagnosis in Belo Horizonte. In compliance with the Brazilian legislation, these animals were recommended for euthanasia, thus it is expected that they have been removed from the environment. However, PMH represents those animals that, although were seropositive in the ELISA test, were negative in the IIFT and therefore were not recommended for euthanasia, and thus remained in the environment as possible reservoirs of *L infantum*. Therefore, they are serologically monitored annually by the Municipal Department of Health. In addition, in the present study we verified through temporal and spatial analyses, that HVL cases are preceded in time by PMH cases, indicating the importance of PMH as a source of vector infection and its relevance, in terms of control, which would justify monitoring of these dogs annually.

In addition to the effect of PCP and PMH on the incidence of HVL, in the present study we observed a temporal relation in the occurrence of VL in an intraspecies way. This result suggests that once HVL or CanL cases have occurred in a region, it is more likely that additional cases of HVL and CanL will occur in this region after a period of time. Despite the low capacity of humans to act as a source of vector infection, the epidemiological characteristics and ecology of the disease may explain this intraspecific relationship. In other words, the disease occurs as a function of the presence of multiple ecological and social determinants, such as the presence of disease vectors, sources of infection, and social vulnerability in an environment, that increase the possibility of infection between hosts that are cohabiting the same environment. Thus, this partially explains the difficulty in controlling VL through the euthanasia of seropositive dogs without considering other ecological and social determinants of the disease, such as those described above. Also, this highlights the importance of the one health approach in the control of this disease in Brazil. Therefore, the assessment of the effectiveness of actions outlined in the SP-VLC and adopted by the healthcare system is important [8, 24].

Araújo et al. [12] showed that the SP-VLC faces challenges in the control of VL because Belo Horizonte is a large territorial area, characterized by variable urban environments, high population and canine densities, and high incidence rate and prevalence of VL. These factors favor dissemination of the disease. Lopes et al. [25] found that 20% of dogs seropositive to *L. infantum* were not euthanized in Belo Horizonte between 1994 and 2007. In addition, approximately 15% of seropositive dogs were not eliminated according to the Information System of the Municipal Health Department of Belo Horizonte (2014). However, these dogs remained in the environment as sources of vector infection with the etiological agent. In our study, we found that 8.79±1.61% on average of seropositive dogs, according to IIFT and ELISA serological tests, were not euthanized between 2006 and 2013.

### Spatial analysis

Understanding the biogeographical complexity of diseases is beneficial for the provision of healthcare services, allowing for the identification of areas with a greater prevalence of such diseases that urgently require implementation of prevention and control programs.

The apparent overlap of areas with high HVL and CanL incidence is the main characteristic found in our study regarding the distribution of cases of CanL and HVL per coverage area of Belo Horizonte. This association was confirmed by means of the mapping of spatial clusters of HVL and CanL cases (*P* < 0.05). The coverage areas located in the South-Central SD are described as examples in our study. These areas showed the lowest PCP, incidence rate, mortality rate and lethality rate over the years. In addition, these areas showed the highest incidence of spatial clusters of low values of CanL (cold spots) (Fig. 3); however, spatial clusters of HVL values were not found during the study period.

An important spatial cluster of CanL obtained for 2007 and 2008 was apparently associated with the clustering of HVL recorded in 2009 (Fig. 3) in the coverage areas located in Venda Nova SD (*P* < 0.05). HVL clustering in Northeast and Northwest SDs, was recorded in 2013, which was preceded by the occurrence of clusters of CanL cases in 2008; that is, the CanL cluster was recorded approximately six years before the occurrence of this cluster of HVL in 2013.

Therefore, our maps showed that HVL and CanL cases were mainly concentrated in the coverage areas located in the SDs of Venda Nova, North, East, Northeast and Northwest, forming a band or area of high incidence risk of CanL cases and, consequently, of HVL cases. Araújo et al. [12] assessed components of the Health Vulnerability Index (HVI) associated with the occurrence of VL to characterize areas of the municipality of Belo Horizonte in respect to social vulnerability. They found that this index may be adequate to identify areas under unfavorable socioeconomic conditions, as well as to describe HVL prevalence in the municipality. Thus, the spatial maps obtained in this study highlighted that the highest HVI were correlated SDs with high incidence of VL in the municipality (Venda Nova, North, East, Northeast and Northwest). In addition, these SDs showed the highest incidence rates of VL over time.

In addition to the socioeconomic factors, variations in the geographical distribution of CanL may occur due to differences in the local ecosystem, specifically regarding the conditions for the development of the vectors. A similar situation has already been discussed by Franco et al. [20] who demonstrated a large variation in the distribution of CanL among European countries between 1971 and 2006. The authors showed that this variation could be explained by climate differences and seasonality associated with the bioclimatic and ecological requirements of the sand flies and other species serving as regional vectors.

Our spatial distribution maps referring to cases of HVL, CanL, and deaths caused by HVL (Fig. 2) demonstrated that the incidence of HVL is apparently associated with the incidence of CanL cases reported in prior years. This pattern is described by both spatial distribution and cluster maps, as it has already been discussed based on VAR analysis and Granger causality testing. Therefore, the spatial distribution suggests that control measures of HVL, when focused on the control of reservoir hosts, may have a significant effect after an extended period of time. This effect is determined by characteristics of the epidemiological chain of the disease, such as long incubation period, and distribution and seasonality of the disease vector.

In the present study, PCP and PMH were complementary in the characterization of dogs that could serve as a source of *L. infantum* infection. In addition, it is important to emphasize the need to understand how the dynamics of euthanasia can affect the proportion of infected animals in the environment, which influence HVL occurrence.

## Conclusions

Our study determined the epidemiological patterns of VL in different geographical regions of Belo Horizonte, State of Minas Gerais, from 2006 to 2013. The temporal distribution of CanL and HVL showed a decline trend; however, no seasonal variation was observed. It was observed that cases of HVL are preceded by PMH and PCP cases. Similar results were observed for intraspecific cases (i.e. between PCP and other canine cases and between HVL and other HVL cases), which indicates the existence of favorable environmental conditions for the transmission and spread of *L. infantum* in Belo Horizonte.

## Abbreviations

ADF: Augmented Dickey-Fuller
AIC: Akaike information criterion
ARIMA: autoregressive integrated moving average
BIC: Bayesian information criterion
CanL: canine leishmaniasis
ELISA: enzyme-linked immunosorbent assay
GECOZ: Zoonosis Control Management
GEEPI: Management of Epidemiology and Information
HQC: Hannan-Quinn criterion
HVI: Health Vulnerability Index
HVL: human viceral leishmaniasis
IIFT: indirect immunofluorescence test
MHD: Municipal Health Department
PCP: proportion of canine seroprevalence
PMH: proportion of monitoring hosts
PRODABEL: Data Processing Company of the Municipality of Belo Horizonte
SCZOO: Information System for Zoonoses Control
SD: sanitary district
SP-VLC: Surveillance Programme and Visceral Leishmaniasis Control
SUS: Unified Health System
VAR: vector autoregressive
VL: viceral leishmaniasis

## References

[1] Brito VN, Oliveira CM, Lazari P, Sousa VRF (2014). Aspectos epidemiológicos da leishmaniose visceral em Jaciara, Estado de Mato Grosso, Brasil, de 2003 a 2012. Braz. J Vet Parasitol..

[2] Alvar J, Velez ID, Bern C, Herrero M, Desjeux P, Cano J (2012). Leishmaniasis worldwide and global estimates of its incidence. PLoS One..

[3] Faucher B, Gaudart J, Faraut F, Pomares C, Mary C (2012). Heterogeneity of environments associated with transmission of visceral leishmaniasis in south-eastern France and implication for control strategies. PLoS Negl Trop Dis..

[4] Argaw D, Mulugeta A, Herrero M, Norbela N, Teklu T (2013). Risk factors for visceral leishmaniasis among residents and migrants in Kafta-Humera, Ethiopia. PLoS Neg Trop Dis..

[5] Belo VS, Werneck GL, Barbosa DS, Simoes TC, Nascimento BW (2013). Factors associated with visceral leishmaniasis in the Americas: asystematic review and meta-analysis. PLoS Negl Trop Dis..

[6] Gramiccia M, Scalone A, Muccio T, Orsini S, Fiorentino E, Gradoni L (2013). The burden of visceral leishmaniasis in Italy from 1982 to 2012: a retrospective analysis of the multi-annual epidemic that occurred from 1989 to 2009. Euro Sur..

[7] Holcman MMM, Sampaio SMP, Rangel O, Casanova C (2013). Spatial and seasonal distribution of *Lutzomyia longipalpis* in Dracena, a city in the western region of the State of São Paulo, Brazil, that is endemic with visceral leishmaniasis. Rev Soc Bras Med Trop..

[8] Ministério da Saúde, Brasil. Manual de controle e vigilância da leishmaniosevisceral. 2014. http://bvsms.saude.gov.br/bvs/publicacoes/manual_vigilancia_controle_leishmaniose_visceral_1edicao.pdf. Accessed 25 Nov 2014.

[9] Coura-vital W, Reis AB, Fausto MA, Leal GGA, Marques MJ (2013). Risk factors for seroconversion by *Leishmania infantum* in a cohort of dogs from an endemic area of Brazil. PLoS One..

[10] Ribas LM, Zaher VL, Shimozako HJ, Massad E (2013). Estimating the optimal control of zoonotic visceral leishmaniasis by the use of a mathematical model. Sci World J..

[11] Ibge. Belo Horizonte. @Cidades, Instituto Brasileiro de Geografia e Estatística. 2014. https://cidades.ibge.gov.br/brasil/mg/belo-horizonte/panorama. Accessed 11 June 2014.

[12] Araújo VEM, Pinheiro LC, Almeida MCM, Menezes FC, Morais MHF, Reis IA (2013). Relative risk of visceral leishmaniasis in Brazil: a spatial analysis in urban area. PLoS Negl Trop Dis..

[13] Morais MH, Fiuza VOP, Araujo VEM, Menezes FC, Carneiro M (2015). Avaliação das atividades de controle da leishmaniose visceral em Belo Horizonte, Minas Gerais, 2006-2011. Epidemiol Serv Saúde..

[14] Morettin PA, Toloi CMC (2006). Análise de séries temporais.

[15] Gujarati D (2006). Econometria básica.

[16] Anselin L (1995). Local Indicators of Spatial Association - LISA. Geo Analysis..

[17] Getis A, Ord JK (1992). The analysis of spatial association by use of distance statistics. Geo Analysis..

[18] Khanmohammadi M, Fallah E, Rahbari S, Sohrabi I, Farshchian M (2010). Seroprevalence of canine visceral leishmaniasis (CVL) in ownership dogs of Sarab, East Azerbaijan, Province, northwest of Iran with indirect immunofluorescence antibody test (IFAT) and its health importance in 2008–2009. J Ani Vet Adv..

[19] Dujardin JC, Campino L, Cañavate C, Dedet JP, Gradoni L (2008). Spread of vector-borne diseases and neglect of leishmaniasis, Europe. Em Infect Dis..

[20] Franco AO, Davies CR, Mylne A, Dedet JP, Gállego M (2011). Predicting the distribution of canine leishmaniasis in western Europe based on environmental variables. Parasitology..

[21] Mestre GLC, Fontes CJF. A expansão da epidemia da leishmaniose visceral no estado de Mato Grosso, 1995–2005. Rev Soc Bras Med Trop. 2007;40:42–8. 10.1590/s0037-86822007000100008 17486252

[22] Colla-Jacques FE, Casanova C, Prado AP (2010). Study of sandfly fauna in an endemic area of American cutaneous leishmaniasis and canine visceral leishmaniasis in the municipality of Espírito Santo do Pinhal, São Paulo, Brazil. Mem Inst Oswaldo Cruz..

[23] Benabid M, Ghrab J, Rhim A, Ben-romdhane R, Aoun K (2017). Temporal dynamics and *Leishmania infantum* infection prevalence of *Phlebotomus perniciosus* (Diptera, Phlebotominae) in highly endemic areas of visceral leishmaniasis in Tunisia. PLoS One..

[24] Camargo-Neves VLF, Katz G, Rodas LAC, Poletto DW, Lage LC, Spinola RMF (2001). Utilização de ferramentas de análise espacial na vigilância epidemiológica de leishmaniose visceral americana - Araçatuba, São Paulo, Brasil, 1998–1999. Cad Saúde Pub..

[25] Lopes EGP, Magalhães DF, Silva JA, Haddad JPA, Moreira EC (2010). Distribuição temporal e espacial da leishmaniose visceral em humanos e cães em Belo Horizonte - MG, 1993 a 2007. Arq Bras Med Vet Zootec..

